# Mifepristone Accelerates HPA Axis Recovery in Secondary Adrenal Insufficiency

**DOI:** 10.1155/2016/4709597

**Published:** 2016-07-19

**Authors:** Pejman Cohan

**Affiliations:** Specialized Endocrine Care Center, 150 North Robertson Boulevard, Suite 210, Beverly Hills, CA 90211, USA

## Abstract

*Context*. Transient secondary adrenal insufficiency (SAI) is an expected complication following successful adenomectomy of ACTH-secreting pituitary adenomas or unilateral adrenalectomy for cortisol-secreting adrenal adenomas. To date, no pharmacological therapy has been shown to hasten recovery of the hypothalamic-pituitary-adrenal (HPA) axis in this clinical scenario.* Case Description*. A 33-year-old woman underwent uncomplicated unilateral adrenalectomy for a 3.7 cm cortisol-secreting adrenal adenoma. Postoperatively, she developed SAI and was placed on hydrocortisone 15 mg/day, given in divided doses. In the ensuing six years, the patient's HPA axis failed to recover and she remained corticosteroid-dependent. Quarterly biochemical testing (after withholding hydrocortisone for 18 hours) consistently yielded undetectable serum cortisol and subnormal plasma ACTH levels. While she was on hydrocortisone 15 mg/day, mifepristone was initiated and gradually titrated to a maintenance dose of 600 mg/day after 5 months. Rapid recovery of the HPA axis was subsequently noted with ACTH rising into the supranormal range at 4 months followed by a subsequent rise in cortisol levels into the normal range. After 6 months, the dose of hydrocortisone and mifepristone was lowered and both were ultimately stopped after 8 months. The HPA axis remains normal after an additional 16 months of follow-up.* Conclusion*. Mifepristone successfully restored the HPA axis in a woman with prolonged secondary adrenal insufficiency (SAI) after adrenalectomy for Cushing's syndrome (CS).

## 1. Introduction

Secondary adrenal insufficiency (SAI) invariably develops following successful adenomectomy of ACTH-secreting pituitary adenomas or unilateral adrenalectomy for cortisol-secreting adrenal adenomas. The abrupt postsurgical transition from a state of cortisol excess to SAI often leads to unpleasant symptoms of cortisol withdrawal including fatigue, nausea, and body aches. During this time, glucocorticoid replacement therapy is often mandatory, particularly during stressful situations. The subsequent recovery of the HPA axis is highly variable and may be influenced by factors such as the duration and severity of preexisting hypercortisolemia, and the dose of glucocorticoid replacement, as well as the underlying etiology of Cushing's syndrome (CS). In a recent retrospective analysis of 230 patients with CS, the median time to HPA axis recovery was 2.5 years, 1.4 years, and 0.6 years for unilateral adrenal, pituitary, and ectopic CS, respectively [1]. Other than physiological glucocorticoid replacement therapy and time, no other treatment has been demonstrated to accelerate the recovery of the HPA axis.

Resumption of normal hypothalamic CRH production appears to be the critical step in HPA axis recovery [2]. As is axiomatic of all endocrine feedback loops, it follows that any treatment that interrupts cortisol negative feedback at the hypothalamic level leads to secondary rises in ACTH and cortisol. Mifepristone is a competitive antagonist of the human glucocorticoid receptor (GR) with an affinity approximately 18 times higher than endogenous cortisol [3]. When administered to healthy human volunteers, mifepristone results in significant dose-dependent increases in plasma ACTH and cortisol levels [4], an effect that is unaccompanied by clinical signs or symptoms of adrenal insufficiency [5]. Mifepristone (Korlym®) is FDA-approved for the treatment of hyperglycemia associated with CS but has never been studied as a treatment to aid HPA axis recovery [6].

Herein, I report a case of woman in whom the addition of mifepristone expedited the recovery of the HPA axis after six years of unsuccessful glucocorticoid replacement therapy.

## 2. Case Report

At age 20, the patient presented with depression for which she was placed on psychotropic medications. At age 28, she developed progressive malaise, 25-pound weight gain, muscle weakness, easing bruising, hypertension, and foot stress fracture. At age 31, DEXA bone density scan disclosed a *T*-score of −3.4 at the lumbar spine and −3.1 at the hip. Further testing for secondary causes of osteoporosis included 24-hour urinary free cortisol which was markedly elevated at 499 mcg/day (range 10−80 mcg/day). Plasma ACTH level was undetectable. Adrenal imaging revealed a right adrenal mass. At age 33, she underwent uncomplicated right laparoscopic adrenalectomy and histopathology confirmed a 3.7 cm adrenocortical adenoma. Postoperatively, the patient was placed on hydrocortisone replacement, initially at a dose of 40 mg/day given in divided doses and gradually tapered to 30 mg/day over the following several months. One year after her adrenalectomy, her body mass index was 20.4 kg/m^2^, and her hydrocortisone had been further tapered to 15 mg daily, which remained her maintenance dose.

During the ensuing six years after her adrenalectomy, the patient generally felt unwell with symptoms of episodic nausea, headaches, lightheadedness, mood swings, and generalized weakness. Attempts to lower the dose of hydrocortisone below 15 mg/day were not tolerated by the patient. Quarterly biochemical testing (after withholding hydrocortisone for 18 hours) consistently yielded undetectable serum cortisol and subnormal plasma ACTH levels. During this 6-year period, she had one pregnancy, occurring 3 years after adrenalectomy and progressing to full-term delivery of a healthy boy. She had two other hospitalizations (separated by 4 years) for near-syncope, malaise, nausea, and vomiting (but without hypoglycemia or hypotension). Both episodes were treated with 48 hours of stress-doses of steroids and intravenous saline, followed by improvement of her symptoms and subsequent tapering of corticosteroids back to a replacement dose of hydrocortisone 15 mg/day.

The failure of the HPA axis to recover six years after adrenalectomy (despite physiological steroid dosing) prompted magnetic resonance imaging of the sella to rule out structural abnormalities of the hypothalamus, infundibulum, and pituitary. This MRI was unremarkable. Other pituitary hormones were also normal. After a detailed discussion of risk and benefit, mifepristone (Korlym) 150 mg every other day was initiated and her dose of hydrocortisone 15 mg/day was continued. Over the subsequent five months, the dose of mifepristone was gradually escalated to 300 mg every other day and then 300 mg daily and finally maintained at 600 mg daily. During this time, rapid recovery of the HPA axis was noted (initially with a rise in ACTH into the supranormal range 4 months after starting mifepristone, followed by a subsequent rise in cortisol levels). After 6 months, the dose of hydrocortisone and mifepristone was lowered and both were ultimately stopped after 8 months. The HPA axis remains normal after an additional 16 months of follow-up.  Table 1 summarizes recovery of the HPA axis after initiation of mifepristone.

The patient tolerated mifepristone remarkably well with the only side effects being amenorrhea and pruritus (she had a preexisting history of idiopathic urticaria). The pruritus was tolerable and managed with over-the-counter antihistamines. At no point during treatment with mifepristone did the patient develop signs or symptoms of adrenal insufficiency. Her menses returned 3 weeks after discontinuation of mifepristone. The HPA axis remains normal after an additional 16 months of follow-up, during which the patient reported marked improvements in sense of well-being and quality of life.

## 3. Discussion

Cushing's syndrome remains one of the most challenging conditions in clinical medicine. Even after accurate diagnosis of hypercortisolemia and tumor localization, successful tumor removal invariably heralds a period of secondary adrenal insufficiency, which renders the patient glucocorticoid-dependent and may take months to years to recover. In a series of 323 patients who underwent successful selective adenomectomies for Cushing's disease, Flitsch et al. reported that 11% remained hypocortisolemic beyond 3 years [7]. In this series, those patients requiring long-term corticosteroid replacement exhibited larger amounts of Crooke's cells in the nonadenomatous pituitary tissue, suggesting that the severity of cortisol excess may be an important determinant in the recovery of the HPA axis. With regard to adrenal Cushing's syndrome, HPA axis recovery time of up to 12 years has been reported in the literature [8]. During this recovery, although the physical and metabolic manifestations of CS gradually regress, sense of well-being and quality of life remain compromised, presumably due to symptoms of cortisol withdrawal and the inherent shortcomings of adrenal replacement therapy.

In the case reported here, the patient's HPA axis showed no sign whatsoever of recovery over a 6-year period after unilateral adrenalectomy. The prolonged suppression of the HPA axis could not be explained by other factors: (1) the patient's maintenance dose of hydrocortisone was limited to only 15 mg/day; (2) stress-dosing of hydrocortisone was limited to only a handful of episodes meriting the stress-dose; (3) the patient was not treated with other glucocorticoids (i.e., transdermal, inhaled, intranasal, intra-articular, or parenteral) or other medications or supplements that could suppress the HPA axis; and (4) cranial MRI did not disclose structural abnormalities of the pituitary and hypothalamus. Even during pregnancy—a state of HPA axis activation—this patient continued to have an undetectable a.m. cortisol level. Only after adding mifepristone, did the HPA axis show signs of improvement. Based on mifepristone's mechanism of action and evidence that resumption of normal hypothalamic CRH activity appears to be the rate-limiting step in HPA axis recovery [2], I propose that central glucocorticoid receptor (GR) blockade deprived the hypothalamus from GR activity, thereby stimulating the CRH-producing neurons of the hypothalamus. Indeed, it has been previously shown that even a single 100 mg dose of mifepristone enhances the ACTH response to CRH in normal volunteers [9]. In this patient, the initial rise of ACTH into the supranormal range followed by the subsequent normalization of cortisol some 2 months later provides indirect support for this hypothesis.

One can argue that simply lowering the dose of hydrocortisone could have yielded similar results. However, innumerable attempts to taper the dose of hydrocortisone below 15 mg/day (even by 2.5 mg increments) failed due to intolerable symptoms of profound fatigue, nausea, and arthralgia. It is therefore noteworthy that the patient did not exhibit signs or symptoms of adrenal insufficiency during the treatment with mifepristone. One explanation may be that mifepristone was introduced gradually starting with a low dose of only 150 mg thrice weekly, while simultaneously maintaining the patient on hydrocortisone replacement. The positive modulatory effects of mifepristone on stress-sensitive regions of the central nervous system (prefrontal cortex and ventral subiculum) may be another potential explanation [10]. Regardless of the possible mechanism/s, this report suggests a differential response when net glucocorticoid exposure is reduced by introducing a GU receptor antagonist as compared to just lowering the dose of steroid replacement.

Although this patient tolerated mifepristone remarkably well, the potential adverse effects of mifepristone deserve mention, particularly for women of reproductive age [11, 12]. Since mifepristone is also a progesterone receptor antagonist, thickening of the endometrial lining can develop, sometimes leading to abnormal vaginal bleeding. The antiprogestational effects will lead to termination of pregnancy. Therefore, pregnancy must be excluded prior to initiation of mifepristone and prevented while being on treatment. The use of mifepristone in patients with CS has also been associated with reversible decreases in high density lipoprotein- (HDL-) cholesterol and mild increases in serum thyroid stimulating hormone (TSH) levels. Other important considerations include drug-drug interactions (mifepristone is both metabolized by and is an inhibitor of CYP3A).

In summary, this case demonstrates the safe and successful use of mifepristone to rapidly restore the HPA axis in a woman with prolonged secondary adrenal insufficiency (SAI) after adrenalectomy for adrenal Cushing's syndrome (CS). The reader is cautioned that this use of mifepristone is off-label and investigational and requires further study. If these findings are replicated in a structured, IRB-approved protocol, then use of mifepristone to restore HPA function may also be potentially extended to the far more prevalent scenario of HPA axis suppression after exogenous glucocorticoid use.

## Figures and Tables

**Table 1:** Time course of HPA axis recovery after initiation of mifepristone.

Month	0	1	3	4	5	6	8	10	11
Mife. dose (mg)	0	150 TIW	300 TIW	300 daily	600 daily	600 daily	300 TIW	0	0
HC dose (mg)	15	15	15	15	15	10	0	0	0
ACTH (pg/mL)	5	15	18	60	78	173	126	26	45
Cortisol (*μ*g/dL)	<1	<1	<1	3.6	4.6	10.5	13.6	13.5	15.3
DHEA (ng/mL)	<0.05	<0.05	<0.05	<0.05	<0.05	<0.05	<0.05	<0.05	0.053

Mife.: mifepristone; HC: hydrocortisone; ACTH: adrenocorticotropic hormone; DHEA: dehydroepiandrosterone; TIW: three times per week.
